# *Médecine tropicale et santé internationale : MTSI* précise sa ligne éditoriale

**DOI:** 10.48327/mtsi.v4i4.2024.618

**Published:** 2024-12-24

**Authors:** Jean-Philippe CHIPPAUX, Jean-Paul BOUTIN, Michel DEVELOUX, Alain EPELBOIN, Pierre GAZIN, François MOUTOU, Jean-François PAYS, Eric PICHARD SFMTSI

**Affiliations:** Institut Pasteur, 25 rue du Dr Roux Paris 75015

**Keywords:** Médecine tropicale, Médecine coloniale, Pathologie exotique, Maladies infectieuses, Régions chaudes, Déséquilibres environnementaux, Déséquilibres socioéconomiques, Mondialisation, Urbanisation, Changement climatique, Pathologies tropicales, Tropical medicine, Colonial medicine, Exotic pathology, Infectious diseases, Hot regions, Environmental inequalities, Socio-economic inequalities, Globalization, Urbanization, Climate change, Tropical pathologies

## Abstract

La médecine tropicale, initialement liée à la médecine coloniale et à la pathologie exotique, se concentrait sur les maladies infectieuses des régions chaudes et sur les déséquilibres environnementaux et socioéconomiques. Les bouleversements mondiaux tels que la mondialisation, l'urbanisation et le changement climatique ont élargi le champ des maladies, avec une émergence de pathologies tropicales dans les régions tempérées et une montée des maladies non transmissibles (traumatiques, métaboliques, psychiatriques, etc.) dans les pays du Sud. La revue *Médecine Tropicale et Santé Internationale (MTSI)* accompagne ce changement de paradigme en intégrant les maladies non transmissibles et en contextualisant les conditions locales d'apparition, de diagnostic et de prise en charge des pathologies. Elle privilégie les analyses basées sur les spécificités locales, incluant les aspects culturels, socioéconomiques et écologiques, ainsi que les contraintes des systèmes de santé.

*MTSI* insiste ainsi sur la contextualisation dans les articles soumis, notamment pour les études originales et les cas cliniques, en insistant sur l'impact des conditions locales, les obstacles diagnostiques et thérapeutiques, et la prise en compte des médecines traditionnelles. Elle invite les auteurs à démontrer la pertinence et la nouveauté de leurs observations tout en respectant les recommandations formelles pour la publication.

## Abstract (en)

Tropical medicine, initially associated with colonial medicine and exotic pathology, focused on infectious diseases of warm regions and on environmental and socioeconomic imbalances. Global upheavals such as globalization, urbanization and climate change have broadened the scope of diseases, with the emergence of tropical pathologies in temperate regions and an increase in non-communicable diseases (traumatic, metabolic, psychiatric, etc.) in southern countries.

*Médecine Tropicale et Santé Internationale (MTSI)* accompanies this paradigm shift by integrating noncommunicable diseases and contextualizing the local conditions in which pathologies occur, are diagnosed and treated. It favors analyses based on local specificities, including cultural, socioeconomic and environmental aspects, as well as health system constraints.

*MTSI* therefore insists on contextualization in submitted articles, especially for original studies and clinical cases, emphasizing the impact of local conditions, diagnostic and therapeutic barriers, and the consideration of traditional medicines. It invites authors to demonstrate the relevance and novelty of their observations, while respecting the formal recommendations for publication.

La médecine tropicale est l'avatar, notamment, de la médecine coloniale et de la pathologie exotique. Ces disciplines ont toutes été fondées sur l'origine géographique. Elles concernaient la santé et les pathologies observées dans les « contrées lointaines », le plus souvent chaudes, ou chez des personnes qui en revenaient. En fonction des circonstances, les différentes dénominations et définitions n’étaient pas exemptes de préjugés politiques et de considérations socioéconomiques. Classiquement, elles traitaient des pathologies infectieuses, avec des modalités de transmission diverses (souvent épidémiques), nutritionnelles ou, plus rarement, génétiques. Les approches soulignaient les particularités de l'environnement (par exemple, le rôle des réservoirs et des vecteurs d'agents pathogènes), les déséquilibres alimentaires (le plus souvent la malnutrition protéino-énergétique), les insuffisances structurelles de l'offre médicale, les conditions de vie, la culture ou les traditions locales. Ces approches ont longtemps expliqué et justifié le cadre plus ou moins consensuel de la médecine tropicale. Des changements majeurs ont apporté un nouvel éclairage à la fin du XX^e^ siècle. L'accélération de la mondialisation, l'urbanisation croissante, la transition démographique (avec le vieillissement de la population), et le changement climatique ont entraîné un brassage inédit des maladies, y compris d'origine animale, et leur émergence dans des lieux où elles n’étaient pas attendues. Des maladies infectieuses et épidémiques d'origine tropicale gagnent les régions tempérées même si elles restent plus prégnantes au Sud. Certes, les villes d’Europe et d’Amérique du Nord ont connu dans le passé des épidémies de maladies tropicales (fièvre jaune par exemple), mais elles étaient éphémères alors qu'aujourd'hui, elles semblent pouvoir s'installer. Parallèlement, les pathologies traumatiques (accidents de la circulation, violences), cancéreuses, psychiatriques, métaboliques et dégénératives se développent au Sud où elles tendent même à remplacer les maladies transmissibles. La diffusion des techniques et compétences médicales dans les pays en développement, même si elle n'est pas équitablement accessible, favorise le diagnostic – sinon la prise en charge – de ces pathologies ubiquitaires. Dès lors, l’étude de ces pathologies désormais observées au Sud peut apparaître originale et pertinente si l'on en montre les spécificités locales permettant de les caractériser et d'en favoriser le contrôle. *Médecine Tropicale et Santé Internationale (MTSI)* souhaite reconnaître et accompagner ce changement de paradigme.

Ce n'est plus seulement la localisation géographique des maladies qui prévaut dans les études épidémiologiques, cliniques et thérapeutiques. S'y ajoute l'ouverture des thématiques aux maladies non transmissibles, c'est-à-dire aux pathologies ou désordres métaboliques, tumoraux, dégénératifs, génétiques ou mentaux. *MTSI* est attentive à la contextualisation qui met en lumière les conditions locales expliquant l'apparition d'une maladie, les particularités de son diagnostic et de sa prise en charge. Il en découle des recommandations appropriées à l'endroit de la population, du personnel de santé et des autorités sanitaires et politiques.

La description d'une pathologie ou d'une observation clinique doit être mise en perspective avec les circonstances de survenue, le parcours de soins, les modalités du diagnostic et de la prise en charge en fonction des moyens disponibles, la qualité de l'offre de soins, les pratiques locales et les difficultés rencontrées pour sa prévention, son dépistage, sa surveillance ou l'application des stratégies de lutte. La rareté ou la nouveauté pour la région considérée d'une maladie – surtout cosmopolite ou bien connue ailleurs – ne justifient pas, à elles seules, la soumission d'un manuscrit à *MTSI.*

Dans une revue systématique, il convient de décrire la situation dans les pays tropicaux ou en développement en expliquant et commentant les différences et les particularités par rapport à celles d'autres pays (fréquence, difficultés diagnostiques ou thérapeutiques, importance socioéconomique, etc.).

Dans une étude originale, il faut préciser les caractéristiques de l'affection dans le pays ou la région en termes épidémiologiques, écologiques et socioéconomiques, l'impact des habitudes et pratiques (notamment culturelles) sur son incidence, sa gravité et son traitement, ainsi que le contexte sécuritaire (conflits armés, terrorisme, risques naturels).

Lors d'une présentation de cas clinique, il est indispensable de détailler l'histoire de la maladie (en particulier le parcours thérapeutique incluant les médecines traditionnelles et alternatives), le motif de consultation, les diagnostics différentiels notamment avec les affections tropicales, les contraintes et limites propres au pays concernant le diagnostic (par exemple le manque de moyens et de formation du personnel, le prix des examens souvent hors de portée du patient) et les problèmes liés à la prise en charge (disponibilité et coût des médicaments, problèmes d'observance, insuffisance de la pharmacovigilance, etc.).

La contextualisation est un élément fondamental de la discussion. Elle décrit et commente les caractéristiques du sujet et de son étude en fonction des moyens accessibles, des circonstances de découverte et de prise en charge. A cet égard, malgré un recours presque systématique aux médecines traditionnelles ou alternatives, ces dernières ne sont habituellement pas documentées. Pourtant, une meilleure connaissance des raisons de leur utilisation et des résultats diagnostiques et thérapeutiques est nécessaire à la prise en compte de leurs mérites et insuffisances, autant qu’à leur intégration dans l'offre de santé. Ainsi, le lecteur qui n'est pas familier avec la pathologie présentée ou avec les conditions locales d'exercice de la médecine, pourra comprendre le déroulé du diagnostic, son résultat, les choix thérapeutiques qui ont été faits et leurs conséquences. Les comparaisons avec d'autres études ne sont d'aucune utilité si elles ne sont pas mises en perspective. Le plus souvent, elles sont à proscrire. Réalisées dans d'autres environnements écologiques, socioéconomiques et politiques, avec des objectifs distincts ou des méthodologies différentes (notamment concernant le recrutement des patients et les critères de décision), elles ne supportent pas le rapprochement sauf si elles sont accompagnées d'explications démontrant leur comparabilité. Nous encourageons les auteurs souhaitant soumettre un manuscrit à *MTSI* à se poser la question des circonstances et des conditions dans lesquelles est menée l'observation qu'ils décrivent, et de la nouveauté qu'elle apporte à ce qui est déjà connu dans ce domaine. Dans tous les cas, il est indispensable de suivre scrupuleusement les recommandations aux auteurs, disponibles en ligne (https://revuemtsi.societe-mtsi.fr/index.php/bspe-articles/information/authors) afin d’éviter tout risque de rejet du manuscrit ou un retard de publication. La revue pourra d'autant mieux aider les auteurs à valoriser leurs travaux, observations et résultats.

