# Supplementary material for: Tropical medicine and international health: MTSI clarifies its editorial line
Source: Med Trop Sante Int. 2024 Dec 24;4(4):mtsi.v4i4.2024.618. [Article in French] doi: 10.48327/mtsi.v4i4.2024.618 (PMC11892381; doi:10.48327/mtsi.v4i4.2024.618)
Supplement: Related texts

# Research article: *Tropical medicine and international health: MTSI* clarifies its editorial line

Tropical medicine is an offshoot of colonial medicine and exotic pathology. These disciplines were all geographically based. They were concerned with the health and pathologies observed in “distant lands,” usually hot ones, or in people returning from them. Depending on the circumstances, the various names and definitions were not free from political prejudices and socio-economic considerations. Classically, they were infectious pathologies with different modes of transmission (often epidemic), nutritional or, more rarely, genetic. These approaches emphasized the particularities of the environment (e.g., the role of pathogen reservoirs and vectors), nutritional imbalances (most often protein-energy malnutrition), structural deficiencies in medical care, living conditions, culture, or local traditions. For a long time, these approaches explained and justified the more or less consensual framework of tropical medicine.

Major changes brought a new perspective at the end of the 20^th^ century. The acceleration of globalization, growing urbanization, demographic change (with an aging population) and climate change have led to an unprecedented mixing of diseases, including those of animal origin, and their emergence in places where they were not expected. Infectious and epidemic diseases of tropical origin are spreading to temperate regions, although they remain more prevalent in the South. In the past, cities in Europe and North America have experienced epidemics of tropical diseases (e.g., yellow fever), but these were short-lived, whereas today they seem to have the potential to become established. At the same time, traumatic pathologies (traffic accidents, violence), cancer, psychiatric, metabolic and degenerative diseases are on the rise in the South, where they are even tending to replace communicable diseases. The spread of medical techniques and skills in developing countries, even if they are not equally accessible, favors the diagnosis, if not the treatment, of these ubiquitous pathologies. Consequently, the study of these pathologies, now observed in the South, can appear original and relevant if we show the local specificities that allow us to characterize them and promote their control.

*Médecine Tropicale et Santé Internationale (MTSI)* wants to recognize and support this paradigm shift.

Epidemiological, clinical and therapeutic studies are no longer based solely on the geographical location of diseases. We are also opening up to non-communicable diseases, i.e. metabolic, tumoral, degenerative, genetic or mental pathologies or disorders. *MTSI* therefore insists on contextualization, which highlights the local conditions that explain the occurrence of a disease and the particularities of its diagnosis and management. This leads to appropriate recommendations for the population, health professionals and health and political authorities.

The description of a pathology or clinical observation must be put into perspective with the circumstances of its occurrence, the care pathway, the diagnostic and management modalities according to the resources available, the quality of the care provided, local practices and the difficulties encountered in its prevention, screening, surveillance or the application of control strategies. The rarity or novelty of a disease in the region under consideration, especially if it is cosmopolitan or well-known elsewhere, does not in itself justify the submission of a manuscript to *MTSI.*

A systematic review should describe the situation in tropical or developing countries, explaining and commenting on differences and peculiarities compared with those in other countries (frequency, diagnostic or therapeutic difficulties, socioeconomic importance, etc.).

An original study should describe the epidemiological, environmental, and socioeconomic characteristics of the condition in the country or region, as well as the impact of habits and practices (especially cultural) on its incidence, severity, and treatment, and the security context (armed conflict, terrorism, natural hazards).

When presenting a clinical case, it is essential to describe in detail the history of the disease (in particular the therapeutic pathway, including traditional and alternative medicines), the reason for consultation, differential diagnoses, particularly for tropical diseases, the constraints and limitations specific to the country in terms of diagnosis (e.g. lack of resources and staff training, the price of tests, which are often unaffordable to the patient) and problems related to management (availability and cost of medicines, compliance problems, inadequate pharmacovigilance, etc.).

Contextualization is a fundamental element of the discussion. It describes and comments on the characteristics of the subject and its study in terms of the resources available and the circumstances of discovery and management. In this respect, despite the almost systematic use of traditional or alternative medicines, they are usually not documented. However, a better understanding of the reasons for their use and of their diagnostic and therapeutic results is essential if their merits and shortcomings are to be taken into account and if they are to be integrated into the health care system. In this way, readers unfamiliar with the pathology presented or with local medical practice conditions will be able to understand the course of the diagnosis, its outcome, the therapeutic choices made, and their consequences. Comparisons with other studies are useless, unless they are put into perspective. In most cases, they should be avoided. Studies conducted in different environmental, socioeconomic, and political settings, with different objectives or different methodologies (especially in terms of patient recruitment and decision criteria), do not bear comparison unless they are accompanied by explanations that demonstrate their comparability.

*MTSI* encourages authors wishing to submit a manuscript to consider the circumstances and conditions under which the observation they are describing was made, and the novelty it brings to what is already known in the field. In all cases, it is essential to follow the recommendations for authors, available online (https://revuemtsi.societe-mtsi.fr/index.php/bspe-articles/information/authors), to avoid any risk of rejection of the manuscript or delay in publication. The journal will then be in a much better position to help authors make the most of their work, observations and results.
